# Impact of frailty on adverse outcomes after radical cystectomy: a systematic review and meta-analysis

**DOI:** 10.3389/fonc.2026.1740552

**Published:** 2026-03-13

**Authors:** Jiayi Fang, Yongqiong Tan

**Affiliations:** Anesthesia Surgery Center-Operating Room, West China Hospital of Sichuan University, Chengdu, Sichuan, China

**Keywords:** bladder cancer, bladder surgery, complications, frail, mortality

## Abstract

**Objective:**

Bladder cancer predominantly affects older adults, with approximately 70% of cases occurring in individuals aged ≥65 years, placing a substantial proportion of patients at increased risk of frailty and age-related comorbidities. However, the impact of frailty on the surgical outcomes of such patients remains unclear. The present systematic review examined the role of frailty in predicting major complications, mortality, readmission, and non-home discharge in patients undergoing radical cystectomy.

**Methods:**

PubMed, Web of Science, Embase, and Scopus were searched up to May 10, 2025, for relevant studies. Random-effects meta-analysis of crude and adjusted data was conducted to compare outcomes between frail and non-frail patients.

**Results:**

Sixteen studies with 55,811 patients were included. Analysis of crude data showed that risk of major complications (OR: 1.09 95% CI: 0.60, 1.98 I^2^ = 84%), all complications (OR: 1.77 95% CI: 0.63, 4.95 I^2^ = 0%), short-term mortality (<1 year) (OR: 2.23 95% CI: 0.63, 7.84 I^2^ = 57%), long-term mortality (>1 year) (OR: 1.81 95% CI: 0.87, 3.80 I^2^ = 71%), and readmission rates (OR: 1.55 95% CI: 0.51, 4.65 I^2^ = 0%) did not differ significantly between frail and non-frail patients. However, meta-analysis of adjusted data showed increased risk of major complications (OR: 2.50 95% CI: 1.59, 3.95 I^2^ = 20%), mortality (OR: 2.12 95% CI: 1.41, 3.18 I^2^ = 88%), and non-home discharge (OR: 4.10 95% CI: 2.07, 8.13 I^2^ = 40%) in frail patients as compared to non-frail patients.

**Conclusions:**

Frailty could be a risk factor for adverse outcomes in patients undergoing radical cystectomy. Further robust studies reporting adjusted data are needed to supplement current conclusions.

**Systematic Review Registration:**

https://www.crd.york.ac.uk/prospero/, identifier CRD420251041804.

## Introduction

Bladder cancer (BC) ranks as the tenth most prevalent cancer globally, with higher incidence in men, for whom it ranks as the sixth most prevalent malignancy (1). Geographical variations are also prevalent with higher incidence rates in the developed world (2). BC primarily affects the elderly (≥65 years), with a median age at diagnosis of 73 years (3). Most patients are considered “frail” upon diagnosis due to the presence of several comorbidities, including smoking, prior radiation therapy, and metabolic problems, all of which are major risk factors for BC (4, 5).

Frailty is a geriatric syndrome defined by reduced physiological reserve and heightened susceptibility to stress, resulting in an increased risk of unfavourable health outcomes, disability, and mortality. Frail individuals exhibit poor nutritional status, limited mobility, decreased physical strength and muscle power, poor endurance, and compromised balance and cognitive function (6, 7). In clinical practice, the identification of frailty is becoming increasingly important, particularly when the disease population is predominantly geriatric, as it has been linked to an elevated risk of falls, disability, hospitalisation, and mortality (8).

Among the available treatment options for BC, radical cystectomy is regarded as the gold standard for patients with muscle-invasive or extremely high-risk non-muscle-invasive bladder cancer (9). Notwithstanding recent surgical breakthroughs in the treatment, a large number of patients undergoing radical cystectomy experience perioperative complications that may result in high perioperative mortality (10). Evidence indicates that frail patients undergoing surgery are at a higher risk of perioperative complications (11). With the increase in global life expectancy, a larger number of frail individuals will require radical cystectomy in the near future (12). Therefore, it is necessary for surgeons to understand the implications of frailty on a morbid surgery like radical cystectomy so that appropriate steps can be undertaken to reduce such risks.

In the past few years, a number of studies have examined the impact of frailty on outcomes of radical cystectomy but with variable results (13–16). Moreover, several of the studies had small sample sizes, which limits strong conclusions (15, 16). To present the best possible evidence, we conducted this systematic review to examine the effect of preoperative frailty on outcomes after radical cystectomy.

## Material and methods

### Registration and eligibility

This PROSPERO-registered (CRD420251041804) review followed the Preferred Reporting Items for Systematic Reviews and Meta-Analyses (PRISMA) guidelines (**Supplementary File 1**) (17) to answer the following PECO research question: In adult patients undergoing radical cystectomy (Population), does the presence of pre-operative frailty (Exposure) result in higher risk of adverse outcomes (Outcomes) as compared to non-frail patients (Comparison)?

The eligibility criteria were also formulated based on PECOS, and were as follows:

Population (P): Adult patients undergoing open or minimally invasive radical cystectomy.
Exposure (E): Pre-operative frailty assessed by any validated tool.
Comparison (C): Absence of frailty.
Outcomes (O): Mortality, complications, readmission, and non-home discharge.
Study design (S): All types of comparative studies.

Exclusion criteria were as follows: 1) Non-human studies, 2) Duplicate data from the same database, 3) absence of a comparative group, and 4) review articles and abstracts.

### Literature search

Two independent reviewers (YT and JF) completed a systematic literature search utilising a mix of free and MeSH-phrases covering the databases of PubMed, Web of Science, Embase, and Scopus up to May 10, 2025. Google Scholar was used to identify studies in gray literature. No language restrictions were imposed. The **Supplementary File 2** displays the relevant searches strategies of the databases. Additionally, reference lists of prior reviews were hand-searched to ensure that no relevant literature was omitted.

Studies were imported into EndNote 20 for deduplication. Two reviewers (YT and JF) subsequently evaluated the titles and abstracts of the remaining articles separately to identify those pertinent to the review. The complete texts of selected studies were evaluated against established inclusion and exclusion criteria to determine final selection. Disputes were settled via dialogue.

### Data extraction

Two reviewers (YT and JF) independently extracted all information from the studies. A pilot data extraction form was prepared and tested. The same was then used to extract the following information from the studies: author, year, location, design, sample size, demographic details, frailty definition, comorbidities, surgical approach, percentage of frail patients, outcomes assessed, covariates adjusted, and follow-up.

The primary outcome was the complication rate. Majority studies used the Clavien-Dindo (CD) classification system, and hence, we identified CD ≥3 grade complications as major complications. Secondary outcomes included mortality, readmissions, and non-home discharge. Mortality data were classified based on follow-up as long-term mortality (>1 year) or short-term mortality (<1 year). Non-home discharge was defined as discharge to any destination other than the patient’s home, including skilled nursing facilities, rehabilitation centres, or long-term care institutions.

### Risk of bias

The Newcastle–Ottawa Scale (NOS) was used to assess the quality of studies in the review (18). The studies were evaluated against criteria for outcome assessment, group comparability, and participant selection. The scale ranged from 1 to 9 stars, with higher scores indicating better study quality. Two reviewers (YT and JF) conducted a risk-of-bias analysis and resolved disagreements through discussion.

### Statistical analysis

Comprehensive Meta-analysis software (version 3) was used for the meta-analysis. A random-effects model was used for all analyses, given the baseline heterogeneity between the studies. Outcome data were reported either in the form of crude values or adjusted odds ratios (OR). Data were therefore pooled separately to generate pooled ORs and 95% confidence intervals for all outcomes. In this review, crude data referred to unadjusted outcome comparisons between frail and non-frail patients, typically derived directly from raw event counts or univariable analyses without controlling for potential confounders. In contrast, adjusted data refer to effect estimates from multivariable regression models reported in individual studies, in which outcomes were adjusted for relevant covariates such as age, sex, comorbidities, tumour characteristics, surgical approach, or perioperative factors.

Heterogeneity between studies was assessed using I^2^ statistic. The Cochrane Handbook (19) classifies between-study heterogeneity as mild (I² < 25%), moderate (I² 25%–75%), or severe (I² > 75%). Publication bias was evaluated using Egger’s test. Funnel plots were also plotted for meta-analyses with at least 5 studies. Leave-one-out meta-analysis was conducted to identify outliers.

## Results

**Figure 1:**
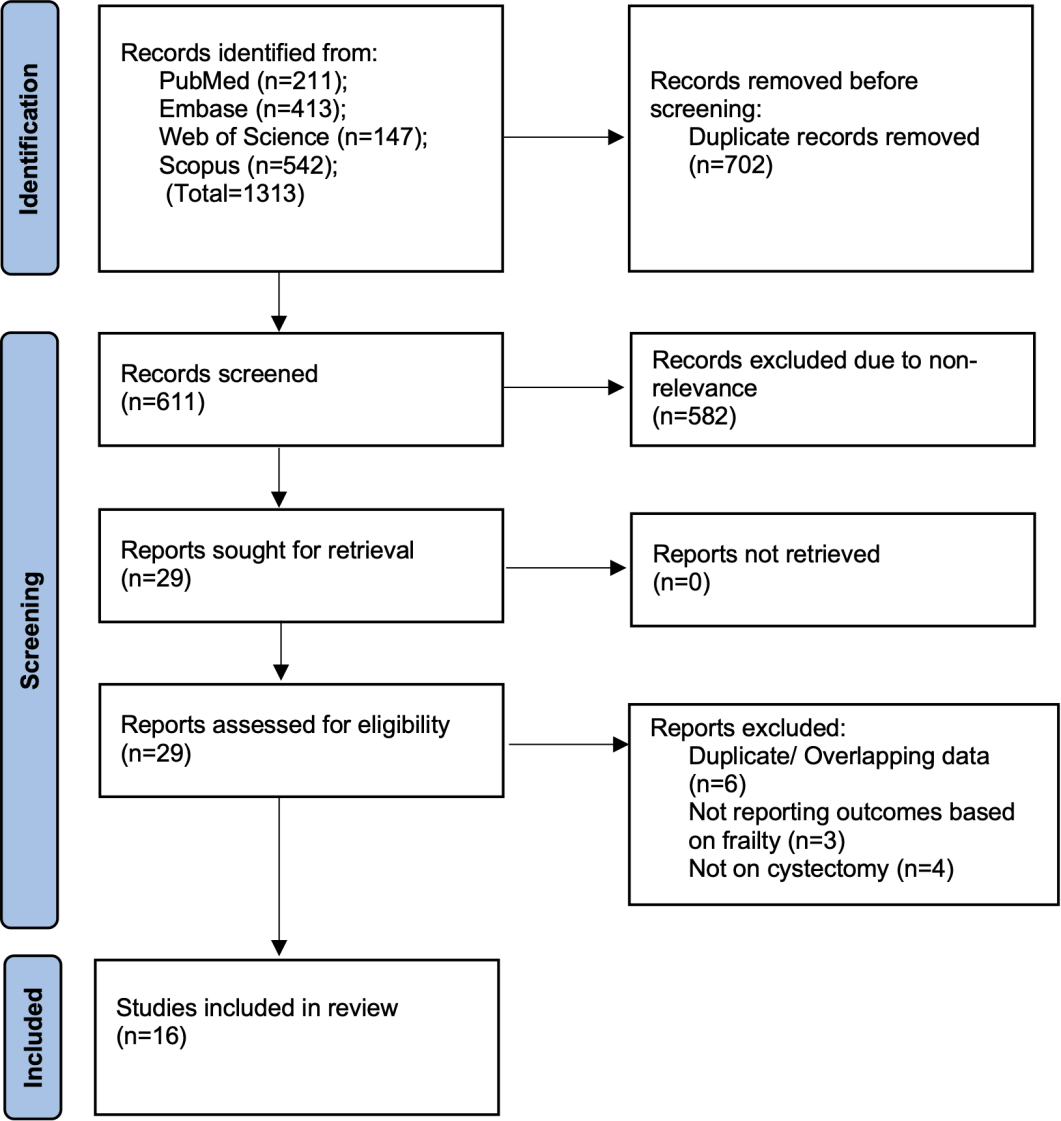
Study flowchart.

**Figure 1** presents the study selection flowchart and the number of records retrieved from each database. A total of 1,313 studies were acquired, and after deduplication, 611 remained. Initial screening of these studies excluded 582 studies as irrelevant. A total of 29 papers were subjected to full-text analysis, of which 16 studies (13–16, 20–31) were included in the review.

### Study details

Study details are shown in **Table 1**. Most studies were retrospective cohort in design, and only three were prospective. Seven studies originated in the USA, two in Germany, two in the Netherlands, and one each in Italy, Greece, Japan, Korea, and Israel. Frailty assessment tools used in the studies included the hospital frailty risk score, simplified frailty index, modified frailty index, Clinical Frailty Scale, chart-derived frailty index, Fried Frailty Criteria, Revised Risk Analysis Index, and the Johns Hopkins Adjusted Clinical Groups Frailty-Defining Diagnoses Indicator. The total sample size of the included studies was 55,811. Majority studies had patients in the elderly age group (≥65 years) with male predominance. Details of comorbidities was not evenly reported by the studies. Tumour, node, metastasis (TNM) stage of patients was not consistently reported by the included studies, and when reported, it was mixed. Five studies did not report the type of radical cystectomy (open or minimally invasive). Three studies included only open cystectomy procedures. In the remaining studies, the percentage of minimally invasive procedures varied from 8.5 to 38%. The percentage of frail patients also varied significantly in the studies, ranging from 2.2 to 76.5%. Majority studies also reported adjusted outcomes but with differences in the adjusted covariates. Only six studies reported long-term outcomes, whereas the others reported only short-term outcomes.

**Table 1:** Details of included studies.

Study	Country	Design	Frailty definition	Sample size	Mean/Median age (years)	Males (%)	DM (%)	CVD (%)	Minimally invasive surgery (%)	Frail patients (%)	Outcomes assessed	Confounders adjusted	Follow-up
Greenberg 2024 (31)	USA	R	Hospital Frailty Risk Score >15	3727	64	100	27.8	35.4	19	25.4	Readmission	DM, CVD, obesity	3 months
Duwe 2024 (30)	Germany	R	simplified Frailty Index ≥2	424	71	77.4	20.3	16.7	10.4	35.8	CD ≥IIIb complications, mortality	Age, BMI, performance status	3 months
Deol 2024 (29)	USA	R	Risk assessment Index-revised ≥21	10191	69	19.7	19.7	0.67	NR	21.6	CD > IV complications, mortality, non-home discharge, readmission, reoperation	Sex, BMI, race, operative time, minimally invasive surgery	1 month
Yu 2023 (28)	Korea	R	Chart-derived frailty index ≥3	1004	68	84	20.8	5.5	NR	9	Mortality	Sex, ASA physical status, comorbidities, tumour stage, grade, neoadjuvant chemotherapy, blood transfusion	1 year
Savin 2023 (27)	Israel	R	modified frailty index ≥2	292	69	77	NR	NR	NR	43.8	CD ≥III complications, mortality	Age, sex, estimated glomerular filtration rate, orthotopic diversion, and neoadjuvant chemotherapy	33 months
Pyrgidis 2023 (26)	Germany	R	Clinical frailty scale ≥5	184	72	71	22	NR	0	51.6	CD ≥III complications, mortality	NR	18 months
Yamashita 2022 (25)	Japan	R	modified frailty index ≥2	238	73	79	16	4	18	22.3	CD ≥III complications, mortality	Age, cancer type, stage, node positivity	38 months
Stamatakos 2022 (16)	Greece	R	Clinical frailty scale ≥4	51	75.6	80.4	NR	NR	0	76.5	Mortality, readmission	NR	1 year
Viles 2020	Netherlands	P	Multiple tools to validated tools to assess physical, mental and social frailty	63	67	76.2	9.5	61.9	38	61.9	CD ≥III complications, mortality, reoperation, readmission, non-home discharge	NR	2 year
Woldu 2019 (24)	USA	R	modified frailty index ≥2	346	67.7	82.4	25.1	NR	18.2	40.8	CD ≥III complications, readmission	NR	3 months
Voskamp 2019	Netherlands	R	modified frailty index ≥2	109	68.8	72.5	14.7	7.3	0	38.4	CD ≥III complications, mortality	NR	3 months
Sathianathen 2019 (22)	USA	R	simplified five-item frailty index ≥3	5516	69	76.7	NR	NR	NR	2.2	CD ≥III complications	NR	NR
Palumbo 2019	USA	R	Johns Hopkins Adjusted Clinical Groups frailty-defining diagnoses indicator	23967	70	79.1	NR	NR	8.5	24.3	All complications, mortality	Age, Charlson comorbidity index, modified frailty index, gender, year of surgery, ethnicity, insurance status, teaching status, hospital volume, region, hospital bed-size, income, lymph node dissection, type or urinary diversion	In-hospital
Michel 2019 (20)	USA	R	Johns Hopkins Adjusted Clinical Groups frailty-defining diagnoses indicator	9459	70	84	NR	NR	23.5	7.1	Non-home discharge	Age, sex, comorbidity score, insurance status	NR
De Nunzio 2019 (14)	Italy	P	simplified five-item frailty index ≥3	117	83	76	NR	NR	NR	50	CD ≥III complications	Age	NR
Burg 2019 (13)	USA	P	Fried Frailty Criteria ≥2	123	74	82.9	NR	NR	33.2	44.9	CD ≥III complications	Urinary diversion	3 months

BMI, body mass index; DM, diabetes mellitus; cardiovascular disease; CD, Clavien-Dindo; RC, radical cystectomy; ASA, American Society of Anesthesiologists; NR, not reported; P, prospective; R, retrospective.

### Risk of bias

**Table 2** shows the NOS scores of the individual studies. All studies received a score of 4 for cohort selection. Six scores were not awarded points for comparability as adjusted data was not reported. Eight studies were awarded 8 or 9 points indicting high quality, while the remaining received six or seven points, indicating medium quality.

**Table 2:** Risk of bias in the included studies.

Study	Selection of cohort	Comparability of groups	Outcome assessment	Total score
Greenberg 2024 (31)	4	2	2	8
Duwe 2024 (30)	4	2	2	8
Deol 2024 (29)	4	2	2	8
Yu 2023 (28)	4	2	2	8
Savin 2023 (27)	4	2	3	9
Pyrgidis 2023 (26)	4	–	3	7
Yamashita 2022 (25)	4	2	3	9
Stamatakos 2022 (16)	4	–	3	7
Viles 2020	4	–	3	7
Woldu 2019 (24)	4	–	2	6
Voskamp 2019	4	–	2	6
Sathianathen 2019 (22)	4	–	2	6
Palumbo 2019	4	2	2	8
Michel 2019 (20)	4	2	2	8
De Nunzio 2019 (14)	4	1	2	7
Burg 2019 (13)	4	1	2	7

### Crude data analysis

**Figure 2:**
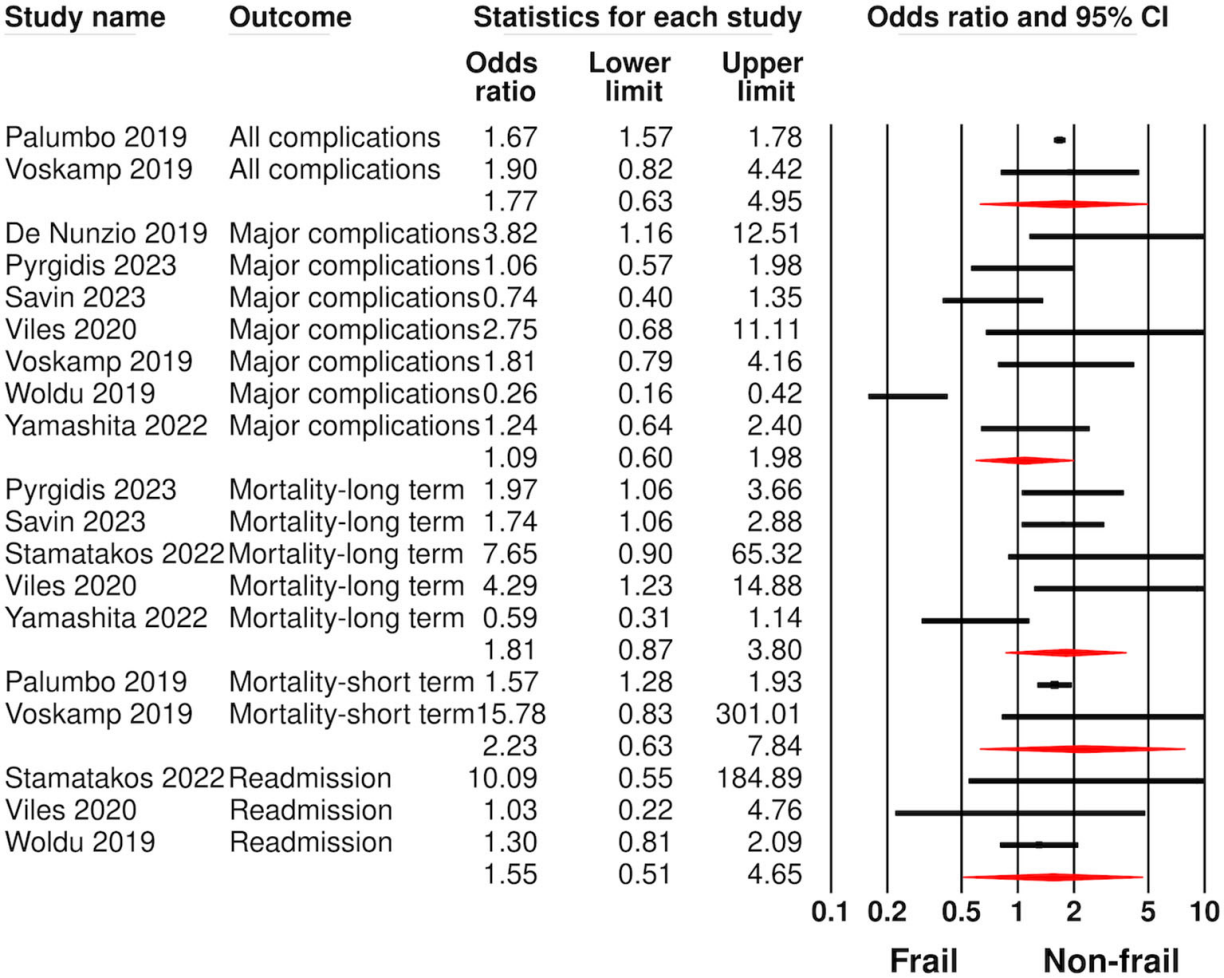
Meta-analysis of complications, mortality, and readmissions between frail and non-frail patients undergoing radical cystectomy using crude data.

Analysis of crude data is presented in **Figure 2**. The risk of major complications was reported in seven studies. Meta-analysis showed no statistically significant difference in the risk of major complications between frail and non-frail patients undergoing radical cystectomy (OR: 1.09 95% CI: 0.60, 1.98 I^2^ = 84%). Likewise, the risk of all complications was also not significantly different between frail and non-frail patients undergoing radical cystectomy (OR: 1.77 95% CI: 0.63, 4.95 I^2^ = 0%).

Five studies reported long-term and two studies reported short-term mortality. Pooled analysis did not show any statistically significant difference in the risk of short-term (OR: 2.23 95% CI: 0.63, 7.84 I^2^ = 57%) or long-term mortality (OR: 1.81 95% CI: 0.87, 3.80 I^2^ = 71%) between frail and non-frail patients. Meta-analysis also showed no significant difference in the risk of readmission between frail and non-frail patients (OR: 1.55 95% CI: 0.51, 4.65 I^2^ = 0%). Lack of data precluded a meta-analysis of non-home discharge with crude data.

Sensitivity analysis results for outcomes with more than three studies is shown in **Supplementary File 3**. The results of major complications and readmissions did not change on exclusion of any study. However, results of long-term mortality turned significant on exclusion of the study of Yamashita et al. (OR: 2.09 95% CI: 1.41, 3.09). None of the outcomes demonstrated publication bias on Egger’s test (p>0.05). Funnel plots for major complications and long-term mortality also failed to demonstrate publication bias (**Supplementary Figures 1**, **2**).

### Adjusted data analysis

**Figure 3:**
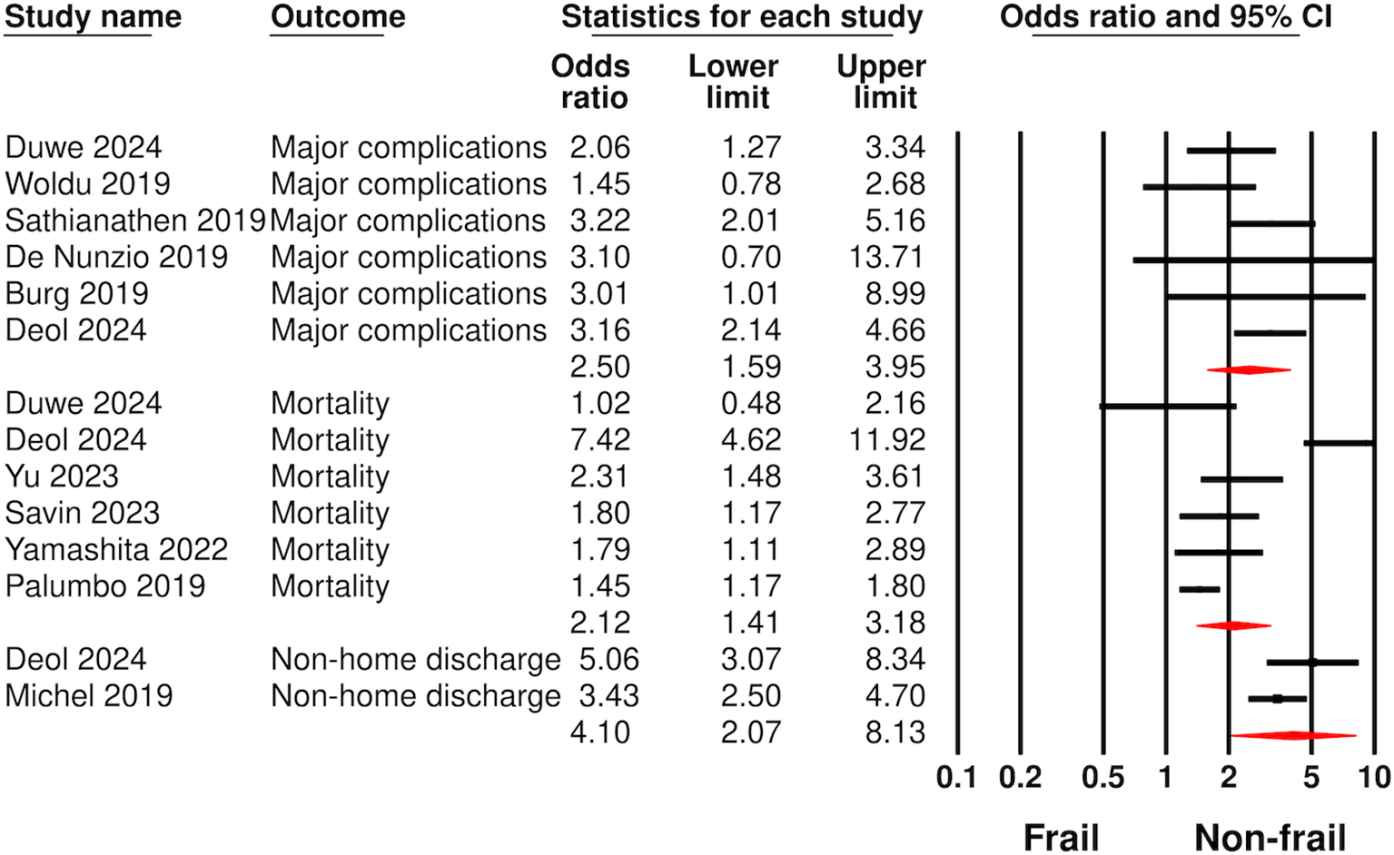
Meta-analysis of complications, mortality, and non-home discharge between frail and non-frail patients undergoing radical cystectomy using adjusted data.

Analysis of adjusted data is presented in **Figure 3**. Six studies reported data on major complications. Meta-analysis showed statistically significant increased risk of major complications in frail as compared to non-frail patients (OR: 2.50 95% CI: 1.59, 3.95 I^2^ = 20%). Six studies reported data on mortality. Mortality data from different follow-up was pooled together for the meta-analysis. Pooled analysis showed statistically significant increased risk of mortality (OR: 2.12 95% CI: 1.41, 3.18 I^2^ = 88%) in frail as compared to non-frail patients. Meta-analysis of just two studies also showed increased risk of non-home discharge (OR: 4.10 95% CI: 2.07, 8.13 I^2^ = 40%) in frail patients. Data for all complications and readmission was insufficient for adjusted analysis.

Sensitivity analysis results for major complications and mortality are shown in **Supplementary File 4**. Both outcomes remained statistically significant on removal of individual studies. None of the outcomes demonstrated publication bias on Egger’s test (p>0.05). Funnel plots for major complications and mortality also failed to demonstrate publication bias (**Supplementary Figures 3**, **4**).

## Discussion

The findings of the present systematic review indicate that frailty may serve as a predictor of negative outcomes in patients undergoing radical cystectomy. Nonetheless, the outcomes differed between the crude and adjusted data analysis. The analysis of crude data indicated a trend towards a higher risk of major complications, short-term mortality, long-term mortality, and readmissions among the frail group, although the results were not statistically significant. Conversely, the frail group exhibited a statistically significant heightened risk of major complications, mortality, and non-home discharge when only the adjusted data were examined. The differences in the results may be due to several factors. First, crude data fail to account for significant confounding variables that could obscure the true relationship between frailty and negative outcomes. Additionally, insufficient adjustment can result in greater variation in crude estimates, stemming from differences in study populations, which may increase inter-study heterogeneity and yield inaccurate estimates. Adjusted analyses can harmonise the variations between studies, resulting in more reliable effect estimates (32). This was particularly noticeable in the meta-analysis of the primary outcome, wherein the I^2^ value was 84% in the crude data analysis but reduced to 20% in the adjusted data analysis. Thirdly, it is also plausible that studies may not have conducted adjusted analysis due to non-significant results of crude data, leading to variations in the current results.

Frailty is characterised by lower functional reserve and increased vulnerability to conditions that disrupt homeostasis, such as surgical stress. It refers to a collection of symptoms or diseases that are responsible for a discernible loss in bodily functions and general endurance. In frail patients, the functional deterioration of multiple organs occurs more rapidly and significantly, while the accumulation of disabilities leads to an overall decline in function (6, 7). In the past, frailty has been recognised as an independent predictor of adverse outcomes in a number of surgical procedures. Panayi et al. (33) in a meta-analysis of 16 studies, including all surgical procedures, found that the modified frailty index strongly correlated with the risk of post-surgical morbidity and mortality. However, it is essential to determine the specific influence of frailty on surgical interventions, given the considerable variability in patient demographics and procedural invasiveness across specialities. Similar to our review, prior studies have also noted frailty to be an important risk factor for adverse outcomes in patients undergoing urological procedures. Aceto et al. (34) in a meta-analysis of 14 studies on both major and minor urological procedures found that frailty predicted adverse outcomes in major urological surgery, but evidence was limited for minor urological procedures. Zheng et al. (35) in their systematic review showed that the presence of preoperative frailty in kidney transplant patients resulted in significantly increased risk of all-cause mortality, delayed graft function, and postoperative complications. A meta-analysis of five cohort studies has also demonstrated that frailty predicts major complications and mortality in patients undergoing both open and robotic prostatectomy (36). A prior systematic review of Ongahi et al. (37) also showed that frailty was a risk factor for adverse outcomes in patients undergoing radical cystectomy, but the authors included multiple studies from the American College of Surgeons-National Surgical Quality Improvement Program database and could not perform a meta-analysis of outcomes. The present review excluded studies with overlapping data and conducted both crude and adjusted analyses to present the best possible evidence.

The current review was unable to distinguish the effects of frailty on open versus robotic procedures. Meta-analysis studies have shown that there may not be any significant difference in major perioperative and oncological outcomes between robotic and open radical cystectomy except for reduced transfusion rates and shorter operative times with the former (38, 39). However, because most studies have used the open incision (extracorporeal) robotic approach, this may have obscured the actual benefits of robotic surgery. Recent data suggests that the technically challenging, intracorporeal robotic reconstruction may lead to lower complication rates and postoperative benefits in patients undergoing radical cystectomy (40). A recent study also shows that robotic surgery may be preferable to open radical cystectomy in frail patients (41), however, further studies are needed for firm evidence.

Frailty assessment remains a debated topic in medical literature. More than 70 instruments have been developed to assess frailty since the initial “Fried Frailty Phenotype” scale was developed in 2001 (42). These assessments are based on physical, psychological, or social components, depending on the individual. Nevertheless, there appears to be a lack of agreement on which scale is considered the gold standard, and the choice of scale is influenced by various factors such as the disease type and simplicity (42, 43). In the present review, both commonly used scales, such as the simplified frailty index, the modified frailty index, and the Clinical Frailty Scale, and less commonly used tools, such as the Johns Hopkins Adjusted Clinical Groups frailty-defining diagnoses indicator, were used. A subgroup analysis was not feasible because of limited data and the extensive variety of tools employed by the included studies. Moreover, there is limited data on the best frailty assessment scale for patients undergoing urological surgery (44). Deol et al. (29) compared the modified frailty index and revised risk analysis index in urologic oncology surgery and found that the latter may have greater utility in predicting adverse outcomes. Nevertheless, further investigation is needed to identify the optimal frailty assessment tool for urological patients.

A number of limitations exist in our meta-analysis. First, not all outcomes were consistently reported across studies. Hence, despite including 16 articles, the number of studies in each meta-analysis remained low. Particularly, for the secondary outcomes, the number of studies was very low to obtain conclusive results. Moreover, most studies reported crude data, and only a limited number reported adjusted data, which limits the strength of our review. Secondly, inter-study heterogeneity was a problem. There were variations in study populations, comorbidity levels, and procedure types (open versus minimally invasive), which may have biased the results. Due to the limited number of studies, a detailed meta-regression was not feasible for these confounding factors. Thirdly, among studies reporting adjusted data, there was a significant difference in the types of confounders adjusted for. None of the studies could include all possible confounders due to data limitations. Moreover, follow-up for mortality varied amongst the included studies, which could have contributed to the inter-study heterogeneity. Likewise, studies also reported different follow-ups for readmission data (both 30-day and 90-day), and a separate analysis was not possible due to limited data. Fourthly, a detailed analysis of specific complications was also not possible due to inadequate data from the included studies. Lastly, while publication bias was assessed using Egger’s test, the small number of studies limits accuracy, and the chance of unpublished negative results cannot be completely ruled out.

This study possesses several significant strengths. To the best of our knowledge, this is the first systematic review and meta-analysis to independently compile crude and adjusted estimates of the influence of frailty on postoperative outcomes following radical cystectomy, thereby facilitating a more precise interpretation of the independent effect of frailty. By excluding overlapping cohorts and aggregating data from more than 55,000 patients, this review provides the most comprehensive quantitative evidence to date. In addition to conventional surgical outcomes, non-home discharge was examined as a patient-centred metric of postoperative functional dependence. Furthermore, adherence to PRISMA guidelines, registration with PROSPERO, and the implementation of extensive sensitivity analyses enhance the methodological rigour of the study.

The present results also have certain clinical implications. Utilising frailty indices and calculators to assess each patient’s health status prior to radical cystectomy can aid surgeons in identifying high-risk candidates. Additionally, it can assist them in modifying specific factors that contribute to patient frailty, enabling them to safely undergo surgery with curative intent. Comprehensive geriatric assessment can help identify reversible contributors to frailty, such as malnutrition, anaemia, functional impairment, and cognitive decline, thereby enabling targeted preoperative optimisation. Multimodal prehabilitation strategies, including nutritional supplementation, structured exercise programs, and psychosocial support, may improve physiological reserve and postoperative resilience in frail patients. In selected individuals with severe frailty or limited life expectancy, consideration of alternative treatment approaches, such as bladder-sparing strategies or palliative intent management, may be appropriate following shared decision-making. Individually tailored multifactorial interventions delivered by a multidisciplinary team comprising physiotherapists, geriatricians, rehabilitation physicians, nurses, and dietitians may enhance frailty status and subsequently improve outcomes following radical cystectomy. There remains a need for randomised controlled trials exploring the impact of such interventions on outcomes of frail patients undergoing radical cystectomy.

## Conclusions

Frailty could be a predictor of adverse outcomes in patients undergoing radical cystectomy. Presence of preoperative frailty can lead to increased risk of major complications, mortality, and non-home discharge. However, present results are supported by a limited number of studies. Further robust studies are needed to supplement current evidence.

## Data Availability

Publicly available datasets were analysed in this study. This data can be found here: PubMed, Web of Science, Embase, and Scopus were searched up to May 10, 2025, for relevant studies.

## Supplementary material

Supplementary Figure 1 Funnel plot for the meta-analysis of major complications (crude data).

Supplementary Figure 2 Funnel plot for the meta-analysis of long-term mortality (crude data).

Supplementary Figure 3 Funnel plot for the meta-analysis of major complications (adjusted data).

Supplementary Figure 4 Funnel plot for the meta-analysis of mortality (adjusted data).
