# Supplementary material for: Impact of frailty on adverse outcomes after radical cystectomy: a systematic review and meta-analysis
Source: Front Oncol. 2026 Mar 13;16:1740552. doi: 10.3389/fonc.2026.1740552 (PMC13021477; doi:10.3389/fonc.2026.1740552)
Supplement: Supplementary file 2 [file DataSheet2.docx]

Supplementary File 2: Search strategy of all databases

| Database | Search strategy |
| --- | --- |
| PubMed | (((((cystectomy) OR (urinary bladder surgery)) OR (bladder resection)) OR (bladder cancer)) AND (((frail) OR (frailty)) OR (geriatric assessment))) AND ((((mortality) OR (complications)) OR (reoperation)) OR (readmission)) |
| Embase | 1. 'radical cystectomy'/exp OR 'radical cystectomy' OR (urinary AND bladder AND surgery) OR (bladder AND resection) OR 'bladder cancer'  2. 'frailty'/exp OR 'frailty' OR frail OR 'geriatric assessment'  3. 'mortality'/exp OR 'mortality' OR 'complication' OR 'hospital readmission' OR 'reoperation'  4. #1 AND #2 AND #3 |
| Scopus | (TITLE-ABS-KEY-AUTH (cystectomy) OR (urinary bladder surgery) OR (bladder resection) OR (bladder cancer)) AND (TITLE-ABS-KEY-AUTH(frail) OR (frailty) OR (geriatric assessment)) AND (TITLE-ABS-KEY-AUTH(mortality) OR (complications) OR (reoperation) OR (readmission)) |
| Web of Science | (((((cystectomy) OR (urinary bladder surgery)) OR (bladder resection)) OR (bladder cancer)) AND (((frail) OR (frailty)) OR (geriatric assessment))) AND ((((mortality) OR (complications)) OR (reoperation)) OR (readmission)) |
