# Supplementary figures and images for: Impact of frailty on adverse outcomes after radical cystectomy: a systematic review and meta-analysis
Source: Front Oncol. 2026 Mar 13;16:1740552. doi: 10.3389/fonc.2026.1740552 (PMC13021477; doi:10.3389/fonc.2026.1740552)

Supplementary File 3: Sensitivity analysis of crude data


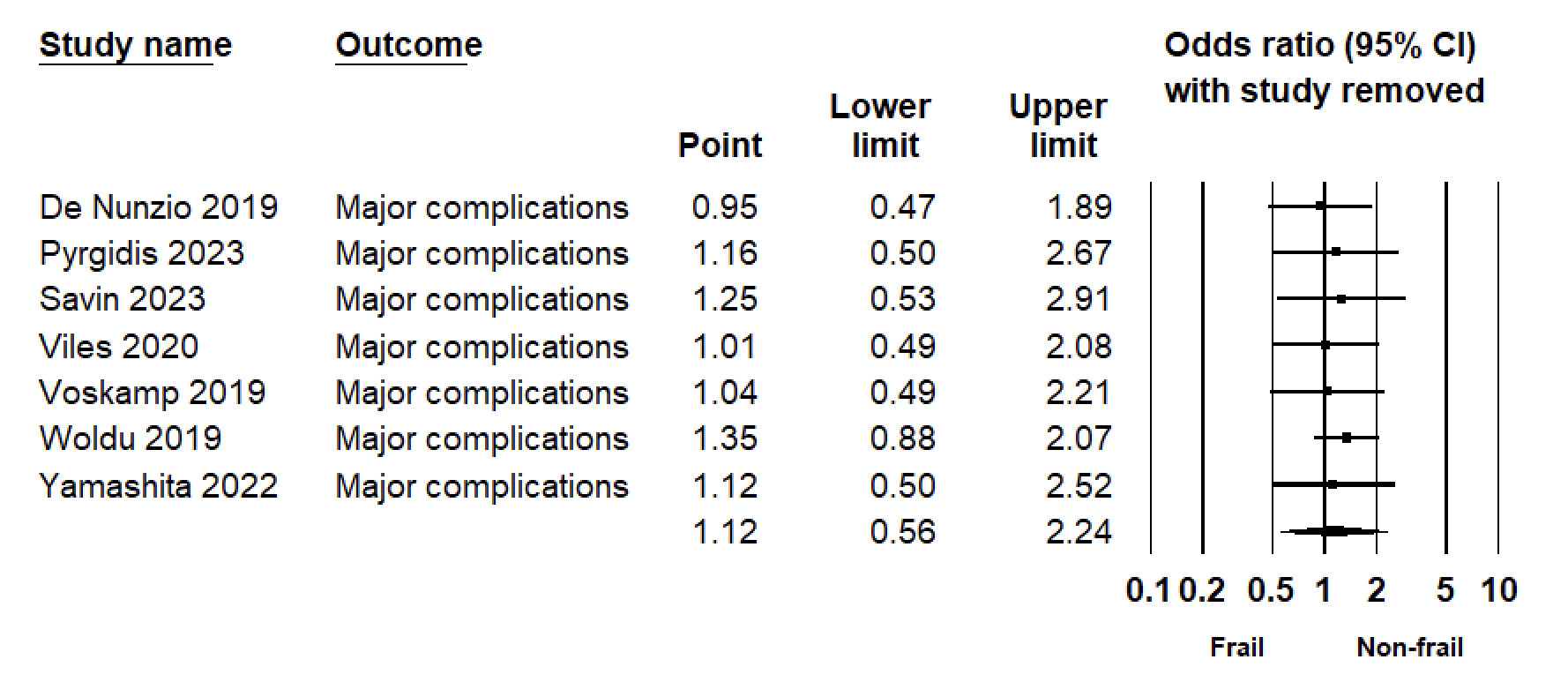


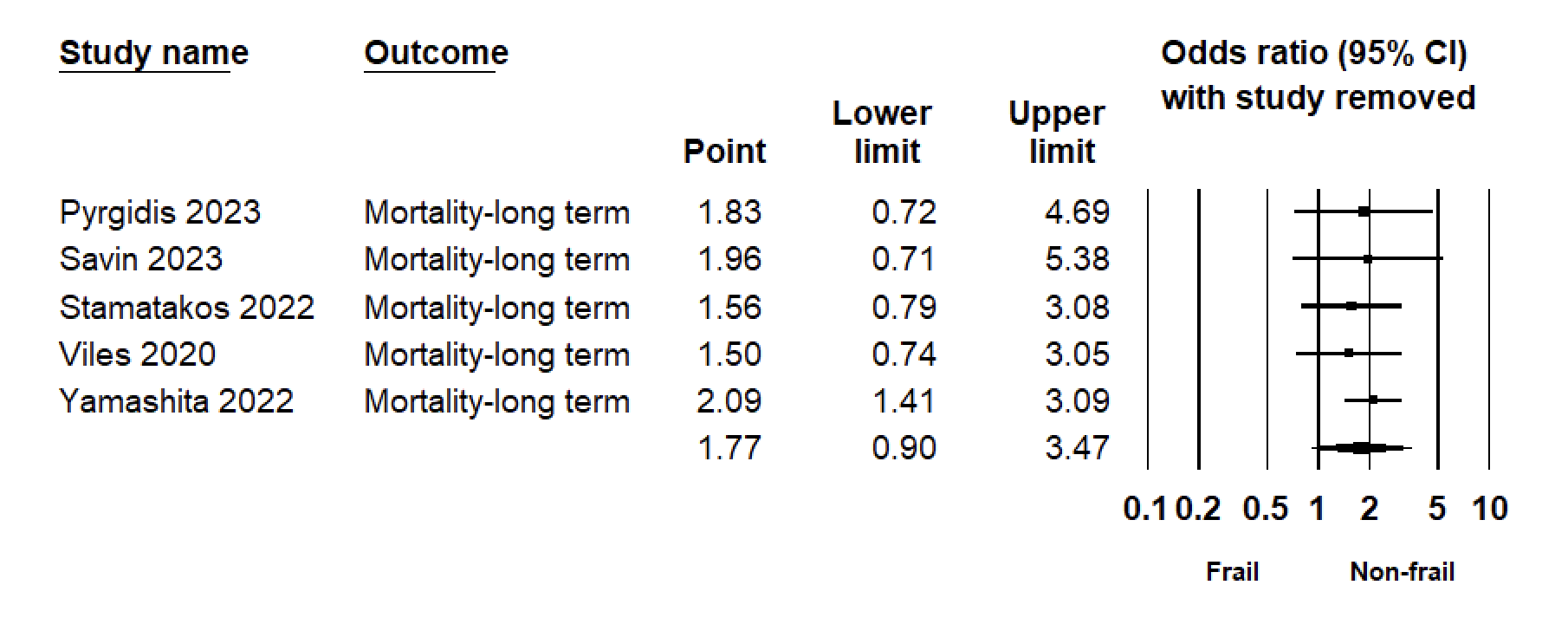


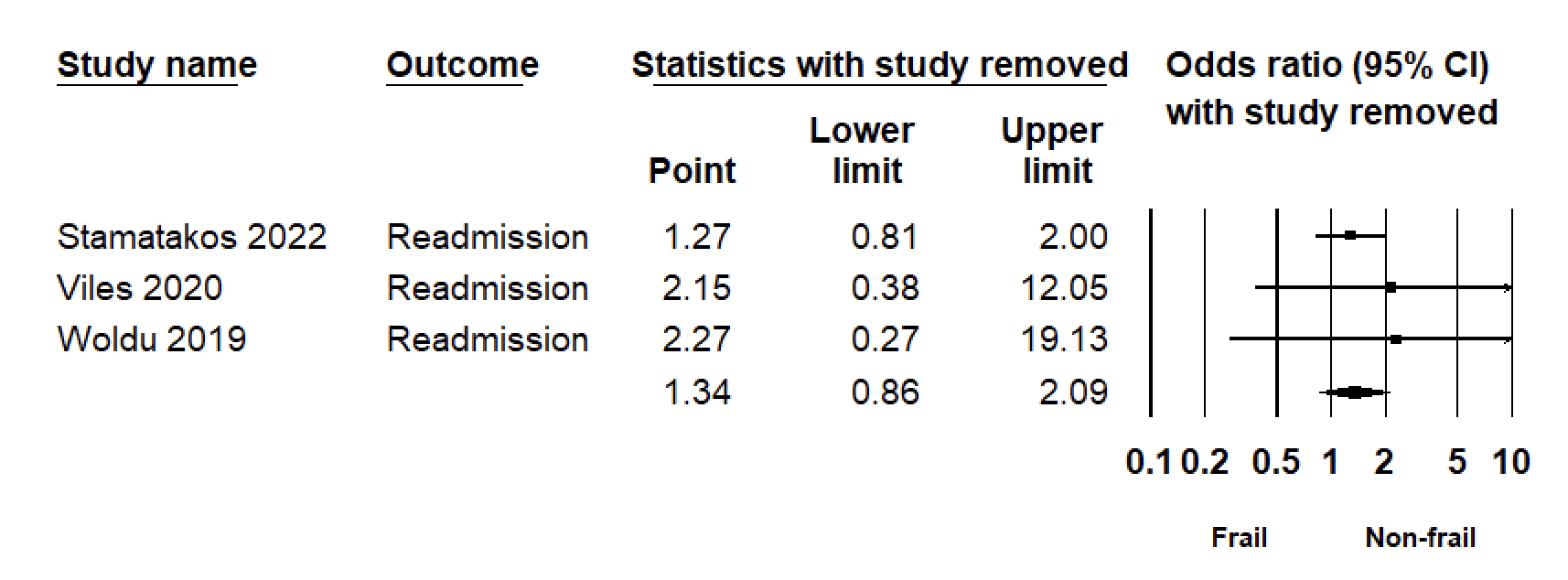

Supplement: Supplementary file 3 [file DataSheet3.docx]

Supplementary File 4: Sensitivity analysis of adjusted data


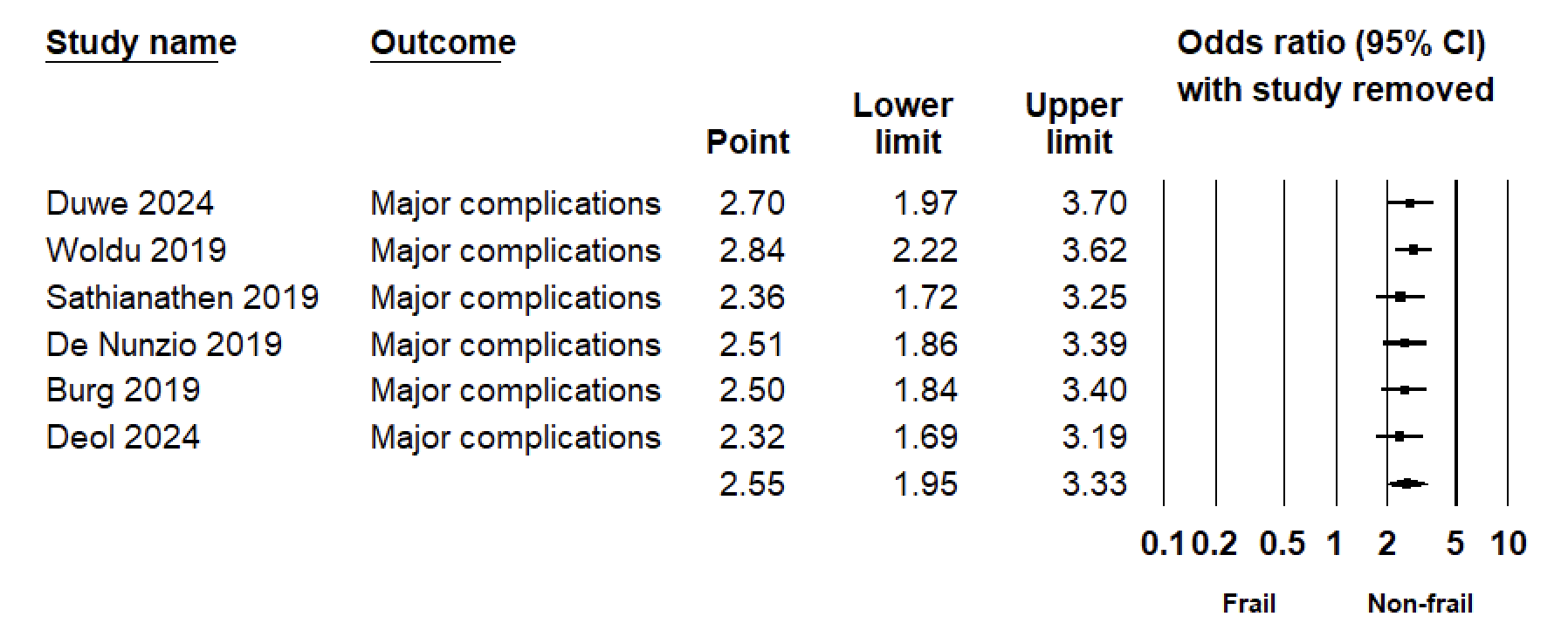


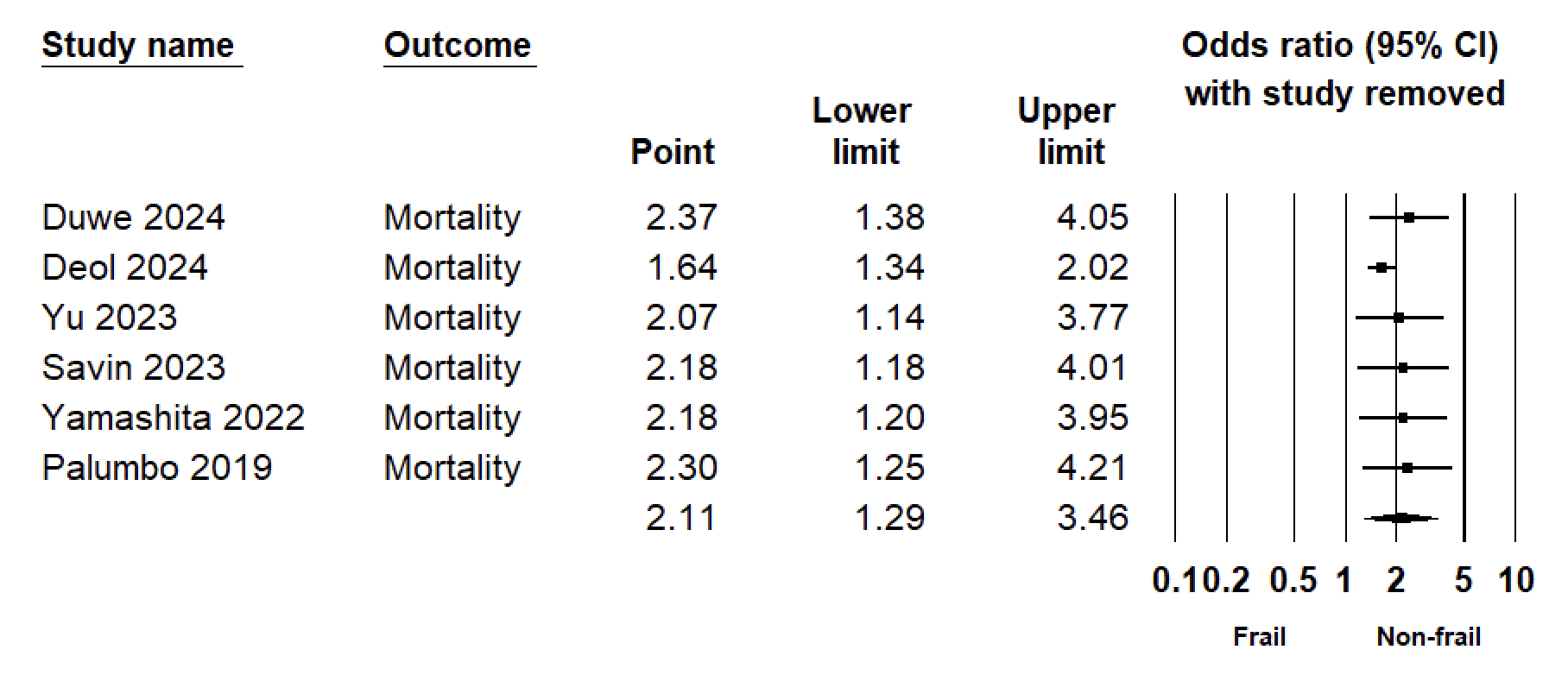

Supplement: Supplementary file 4 [file DataSheet4.docx]

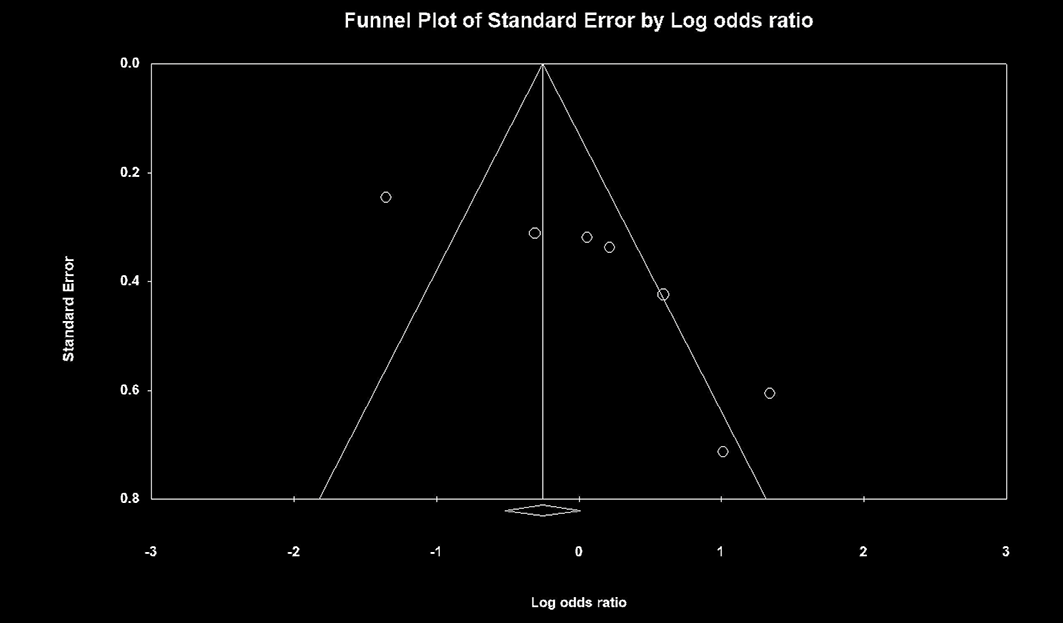

Supplement: Supplementary Figure 1 — Funnel plot for the meta-analysis of major complications (crude data). [file Image1.tif]

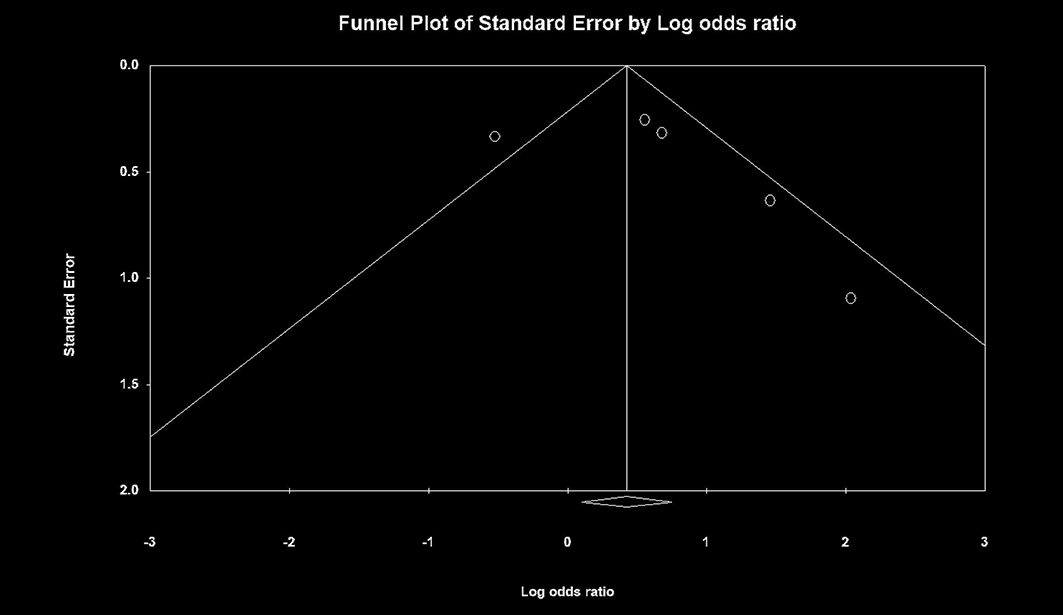

Supplement: Supplementary Figure 2 — Funnel plot for the meta-analysis of long-term mortality (crude data). [file Image2.tif]

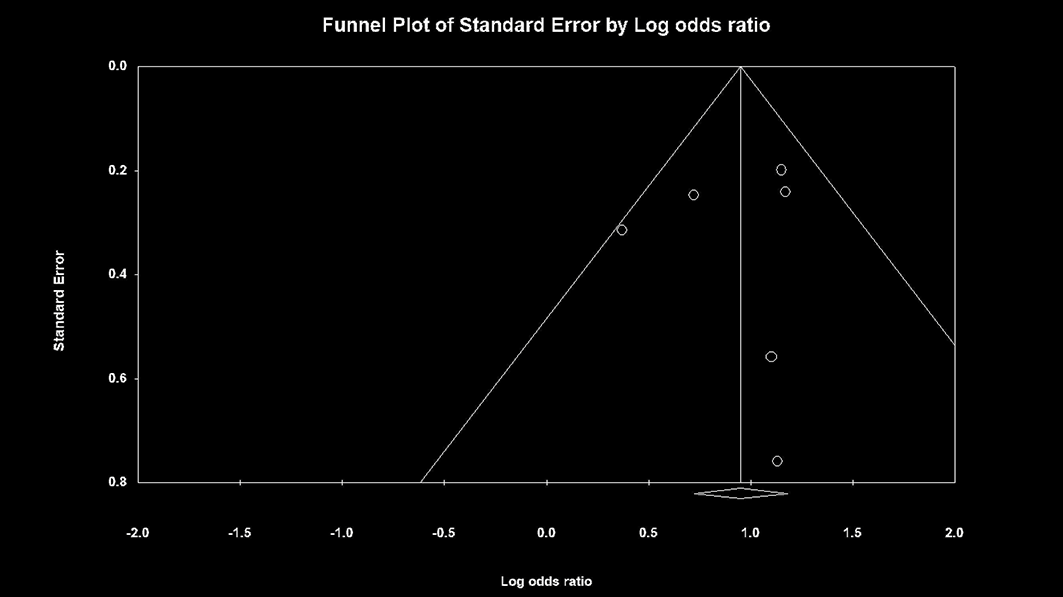

Supplement: Supplementary Figure 3 — Funnel plot for the meta-analysis of major complications (adjusted data). [file Image3.tif]

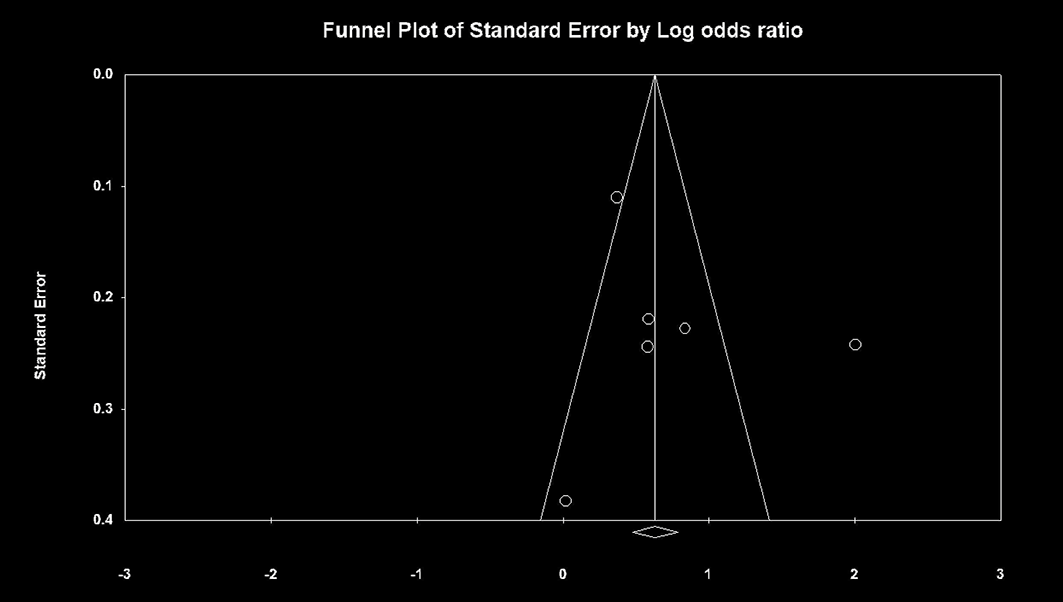

Supplement: Supplementary Figure 4 — Funnel plot for the meta-analysis of mortality (adjusted data). [file Image4.tif]
